# Effect of the Dietary Approaches to Stop Hypertension (DASH) diet on the development of preeclampsia and metabolic outcomes in pregnant women with pre-existing diabetes mellitus: a randomised, controlled, single-blind trial

**DOI:** 10.1017/jns.2023.54

**Published:** 2023-07-06

**Authors:** Gabriella P. Belfort, Patricia C. de Padilha, Dayana R. Farias, Letícia B. G. da Silva, Karina dos Santos, Erlaine de S. Gomes, Thaissa S. V. Lima, Rita Bernardete R. G. Bornia, Karina B. C. Rezende, Claudia Saunders

**Affiliations:** 1 Josué de Castro Institute of Nutrition, Federal University of Rio de Janeiro, 373, Carlos Chagas Filho Ave, University City, Rio de Janeiro, RJ 21941-590, Brazil; 2 Applied Nutrition Department, Federal University of the State of Rio de Janeiro, 296, Pasteur Ave, Rio de Janeiro, RJ 22290-240, Brazil; 3 Social and Applied Nutrition Department, Josué de Castro Institute of Nutrition, Federal University of Rio de Janeiro, 373, Carlos Chagas Filho Ave, University City, Rio de Janeiro, RJ 21941-590, Brazil; 4 Public Health Nutrition Department, Federal University of the State of Rio de Janeiro, 296, Pasteur Ave, Rio de Janeiro, RJ 22290-240, Brazil; 5 Maternity School of the Federal University of Rio de Janeiro, 180 Laranjeiras St, Rio de Janeiro, RJ, 22240-003, Brazil

**Keywords:** Diabetes mellitus, Hypertension, Oxidative stress, Preeclampsia, Pregnancy-induced, DASH, Dietary Approaches to Stop Hypertension

## Abstract

Preeclampsia (PE) affects up to five times more women with pre-existing diabetes mellitus (PDM) than women without it. The present study aimed to identify the effect of the DASH diet on PE incidence (primary outcome) and blood pressure, glycated haemoglobin (GH), serum lipids, glutathione peroxidase (GP), C-reactive protein (CRP – secondary outcomes) in pregnant with PDM. This randomised, controlled, single-blind trial studied sixty-eight pregnant women with PDM throughout prenatal care until delivery (18 weeks) at a public maternity hospital, Brazil. The standard diet group (SDG) received a diet containing 45–65 % carbohydrates, 15–20 % protein and 25–30 % lipids. The DASH diet group (DDG) received the adapted DASH diet with a similar macronutrient distribution, but with a higher concentration of fibres, unsaturated fats, calcium, magnesium and potassium as well as lower saturated fat. Student's *t*, Mann–Whitney *U* and the Chi-square tests were used to compare outcomes. PE incidence was 22⋅9 % in the SDG and 12⋅1 % in the DDG (*P* = 0⋅25). GP levels significantly increased in the DDG (intra-group analysis; mean difference = 1588 [CI 181, 2994], *P* = 0⋅03) and tended to be different from the variation in the SDG (mean difference = −29⋅5 [CI −1305; 1⋅365]; *v*. DDG: 1588 [CI 181; 2994], *P* = 0⋅09). GH levels decreased significantly and similarly between groups (SDG: −0⋅61 [CI −0⋅26, −0⋅96], *P* = 0⋅00) *v*. DDG: −1⋅1 [CI −0⋅57, −1⋅62], *P* = 0⋅00). There was no evidence of a difference in PE incidence at the end of the intervention between the two diets. The DASH diet seems to favour PE-related biochemical markers.

## Introduction

Gestational hypertensive syndromes (GHS) affect about 14 % of pregnancies worldwide. In Latin America and the Caribbean, they are responsible for 22 % of maternal deaths^(1)^. In particular, preeclampsia (PE) has the greatest impact on maternal morbidity and mortality, affecting an estimated 2–8 % of pregnancies worldwide^(2–4)^. In Brazil, it is the leading cause of elective prematurity^(2)^. Its pathophysiology is not yet fully understood, but in recent decades, the influence of oxidative stress and genetic polymorphisms on the genesis of this disorder, which most often affects nulliparous women, has been investigated^(4,5)^. The rs8068318 polymorphism of T-box transcription factor 2 (TBX2), a gene that regulates the transcriptional activity of several other genes, was associated with the highest risk for developing PE in a study with 548 Russian women^(4,5)^. Higher serum concentrations of lipids and C-reactive protein (CRP) are also associated with PE^(6)^.

Women with pre-existing diabetes mellitus (PDM), either type 1 or type 2, are considered to be at a higher risk of developing PE. With the increased prevalence of diabetes mellitus (DM) observed in recent years, the proportion of pregnant women with PE is also on the rise^(4,7,8)^. The higher number of women with DM, especially type 2, is associated with a growing prevalence of obesity in women of reproductive age^(8)^. Decompensated DM in pregnancy may be also associated with macrosomia, foetal death and long-term consequences for the foetus, such as a higher chance of developing type 2 diabetes mellitus (T2DM) and obesity in adulthood^(8,9)^.

The American Diabetes Association^(8)^ and the Brazilian Diabetes Society^(7)^ suggest that Mediterranean and DASH diets may have a positive impact on blood glucose and blood pressure control in patients with hypertension or diabetes. Given the proven effect of the DASH diet in reducing blood pressure in hypertensive adults and its low glycaemic index and high antioxidant content, this diet could potentially also improve glycaemic control in individuals with or without DM and reduce the risk of disease in adults, such as episodes of acute myocardial infarction^(10–13)^. According to the Brazilian Diabetes Society^(7)^, eating more fruits and vegetables can provide better combinations of antioxidants and micronutrients than taking supplements and is also safer in the long run.

In addition, the DASH diet seems to help reduce the blood pressure, body weight loss and lipid profile of women of reproductive age, besides having a protective effect against the development of gestational DM^(14,15)^. Two studies were conducted in Iran with fifty-two women with gestational diabetes mellitus (GDM) to test the effect of the DASH diet for 4 weeks on perinatal outcomes, comparing it to a control diet^(16,17)^. The group that followed the DASH diet was found to have a lower proportion of caesarean deliveries, less need for insulin use, a lower proportion of macrosomia, lower fasting glucose values, lower serum insulin concentrations and lower HOMA-IR, in addition to having blood that exhibited higher antioxidant capacity. Some more recent studies investigating the effect of the DASH diet on pregnancy have found that it provides better control of blood glucose and weight gain, besides having a protective effect against the development of PE in healthy women^(18–20)^.

Given the scientific evidence of the protective effect of the DASH diet on inflammatory markers, blood pressure and lipid levels in adult populations, the primary aim of this study was to evaluate the effect of the DASH diet adapted to Brazilian women with PDM on the PE incidence, and its secondary aim was to assess its effect on maternal metabolic outcomes (blood pressure, glycated haemoglobin, serum lipids, glutathione peroxidase, CRP).

## Experimental methods

### Design

The study was a randomised, controlled, single-blind, two-arm treatment trial. The women were not told which treatment group they were allotted to. The study protocol was registered on the Brazilian Clinical Trials Registration Platform (Rebec – RBR-4tbgv6).

The study was conducted between November 2016 and March 2020 at the maternity teaching hospital of the Federal University of Rio de Janeiro (MT/UFRJ), Brazil. MT/UFRJ is a reference in Rio de Janeiro for the prenatal care of women with PDM. Pregnant women diagnosed with DM are referred to it from primary care centres through an online system (SISREG).

**Fig. 1.**
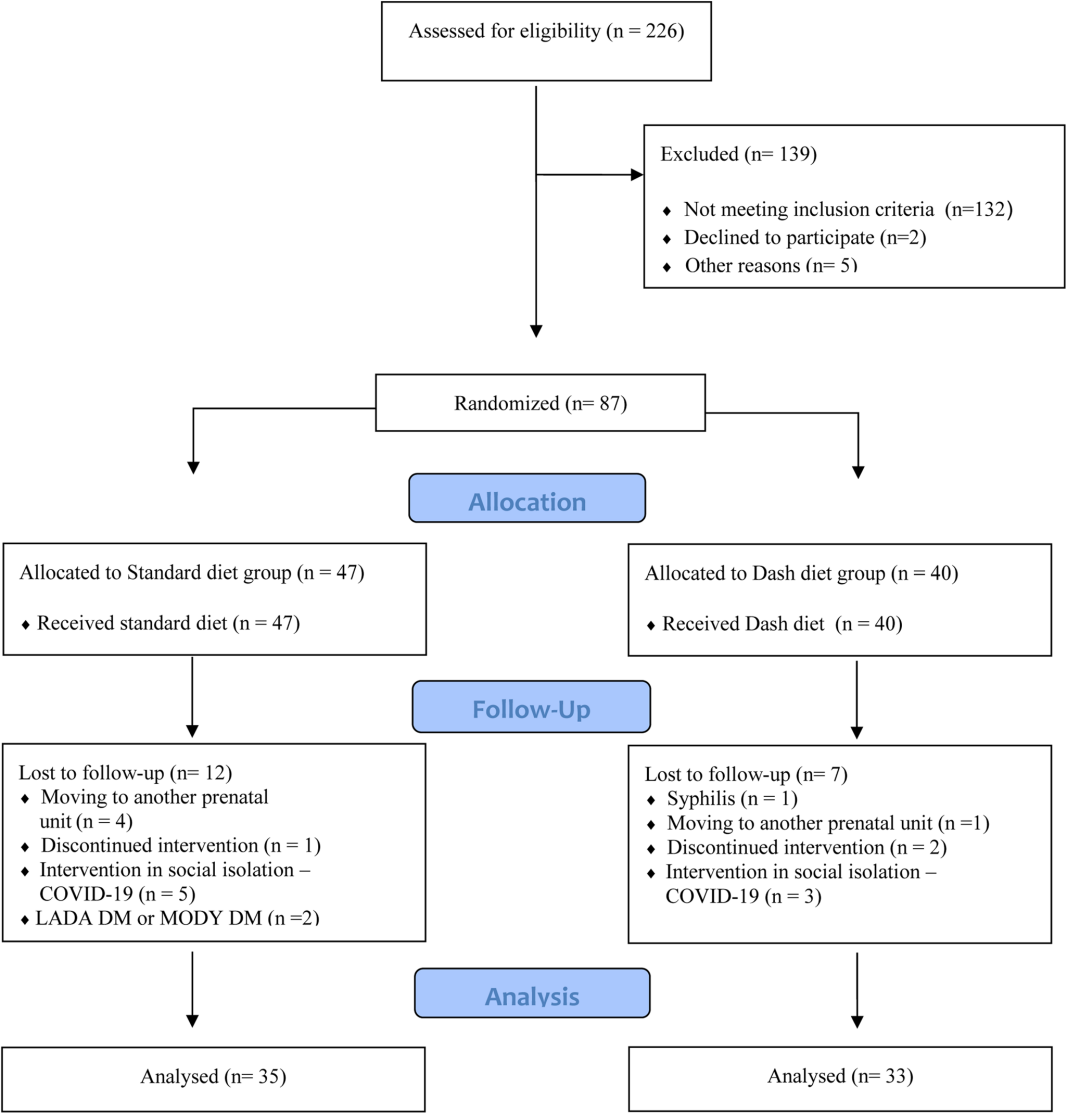
Summary of patient enrolment and follow-up.

Eighty-seven pregnant women with PDM were recruited to participate in the study, and those who accepted and met the inclusion criteria were randomly allocated to the study groups: standard diet group (SDG) or adapted DASH diet (DDG) (Fig. 1).

### Participants

The study population consisted of pregnant women diagnosed with PDM before referral, whose diagnosis was confirmed by an endocrinologist at the study site according to the criteria of the Brazilian Society of Diabetes^(7)^. Those who met the following inclusion criteria were eligible: age ≥18 years at conception; diagnosis of type 1 diabetes mellitus (T1DM) or T2DM with onset before pregnancy (forms of PDM considered for the study); singleton pregnancy; gestational age ≤28 weeks; non-smoker and non-drinker. Women with chronic systemic arterial hypertension were included, but only if it was mild and controlled (systolic blood pressure ≥140 and <160 mmHg and/or diastolic blood pressure ≥90 and ≤110 mmHg) and the women had no diagnosis of PE, eclampsia or HELLP syndrome (haemolysis; elevated liver enzyme levels and low platelet levels) at enrolment. Women with treated and controlled hypothyroidism were also included. Exclusion criteria were: DM complications such as nephropathy, retinopathy, heart disease liver disease or eating disorders.

All the women received prenatal care throughout pregnancy from a multidisciplinary team at MT/UFRJ. Data were collected from the pregnancy, postpartum and newborn medical records and in face-to-face interviews with a nutritionist. All data were collected by a trained and supervised research team.

Insulin doses for both study groups were set by the endocrinologist based on the patients’ gestational weight. In addition, the women were instructed to self-monitor their blood glucose by measuring capillary blood glucose at least six times a day, before and after meals. Finally, all the women who began prenatal care before 16 weeks of gestation were prescribed a daily dose of 100 mg aspirin. All three practices are part of the routine prenatal care offered at the study site.

### Randomisation

Upon enrolment, the women were randomly allocated to either the SDG or the DDG using a random number from a list prepared using Excel® 2007. The women who received an odd number were put in the SDG and the women with an even number were put in the DDG by the nutritionist. The pregnant women did not know which study group they belonged to; only the researcher responsible for providing the nutritional guidance had this information.

### Nutritional intervention

The nutritional intervention for both groups occurred from enrolment to the end of pregnancy and included a minimum of six individual appointments with the nutritionist when individualised guidance was given according to the occurrence of maternal complications. To improve dietary adherence, the women from the DDG were given a portion of seeds (200 g), nuts (150 g) and fat-free milk (280 g) at each visit, while the women from the SDG received a portion of oats (250 g) and low-fat milk (1–2 %, 300 g). All the women were also given a 500 ml bottle of extra-virgin olive oil at their first appointment.

In the preparation of the dietary plans, two methods of dietary guidance were used – the traditional method and the carbohydrate counting method – which the women themselves selected according to their preference^(7)^. Both methods of dietary guidance are associated with good glycaemic control in women with GDM^(21)^.

The total energy value of each diet was calculated individually to promote adequate gestational weight gain. The proportion of macronutrients was similar for the two groups, ranging from 45 to 55 % carbohydrates, 15 to 20 % proteins and 25 to 30 % lipids^(22)^. The energy distribution per meal for both groups was as follows: breakfast 10–15 %, mid-morning snack 5–10 %, lunch 20–30 %, afternoon snack 10–15 %, dinner 20–30 % and supper 5–10 %^(21,23)^.

All the women with low calcium intake (<900 mg/d) were prescribed 500 mg calcium carbonate supplementation per day from the 20th gestational week onwards^(24)^. Sodium intake was limited to 2400 mg/d and both groups were advised against consuming sucrose^(25,26)^.

### Standard diet

The standard diet was the diet already recommended in routine prenatal nutritional care at the study site, which is consistent with American Diabetes Association guidelines^(8,22,23)^. The dietary plan was divided into five to six meals a day at regular times. The women received the diet and a food substitution list, according to food groups (fruits, bread, dairy products, meats, cereals, legumes, fats and vegetables). This plan was based on foods with standard fat content, except for milk, which could be full-fat or semi-skimmed (1–2 % fat). As for cereals and bread, no requirement for wholegrain options was made. In addition, the women also received general nutritional guidelines as part of the standard prenatal nutritional care given at the study site.

### Adapted DASH diet

The DASH diet used in the study was the version translated into Portuguese and adapted to the Brazilian population by Saunders *et al.*^(26)^ and includes the recommended dietary guidelines for this population^(27)^. The dietary plan was divided into five to six meals a day at regular times and the women were also given a list of food substitutes in accordance with the DASH recommendations. The difference between the diets consisted in the fact that the adapted DASH diet proposes: the consumption of whole grain, bread and cereals, fat-free or low-fat dairy products, and a daily portion of seeds and nuts. It contains illustrated nutritional guidelines to encourage the consumption of foods with high concentrations of fibre, potassium, magnesium and calcium.

A comparison of the constituents of the standard and DASH diets applied in the present research was published in the study of Fagherazzi *et al.*^(28)^.

### Anthropometric assessment

Weight measurements (kg) were taken at all the appointments using an electronic platform scale, and height (m) was measured using a stadiometer attached to the scale. Measurements were taken according to standard nursing practice at the study site^(29)^. Pre-pregnancy nutritional status was classified according to pre-gestational body mass index at the first consultation, based on weight measured up to the 14th gestational week or self-reported pre-pregnancy weight, on which weekly and total weight gain was estimated^(30)^. Total weight gain during intervention was calculated as the difference between prepartum weight or weight at the last appointment and weight at the first prenatal nutritional appointment^(29,30)^.

### Assessment of food consumption and dietary adherence

Food intake was assessed in two 24 h recalls administered during the 3rd (between the 22nd and 24th gestational week) and 5th (between the 29th and 34th gestational week) nutritional appointments. Foods in the 24 h recalls were expressed in household measurements, which were then quantified in grams or millilitres per day using a conversion table^(31)^. A Microsoft Excel spreadsheet was used to estimate energy and macro- and micronutrient intake. This included foods and their nutrient profiles according to the United States Department of Agriculture data on the chemical composition of foods^(32)^, the Institute of Nutrition of Central America and Panama food composition tables^(33)^ and the Brazilian food composition table^(34)^.

Adherence to the diets was assessed by reported food intake and weekly weight gain^(35)^. The criteria for this were (1) the amount of food ingested; (2) the quality of the food (frequency of ingestion of food groups); (3) the pattern of meals (the number and times of meals, their composition and any food substitutions) and (4) the adequacy of weight gain about the previous appointment. Weight gain was considered adequate when the woman gained up to 20 % over or below the recommended weight. Adherence was then classified into poor (for women who met only one criterion), good (for women who met two or three criteria) and optimal (for women who met all four criteria).

### Biochemical assessment

Venous blood samples (10 ml) were collected after 8–12 h fasting at baseline and after 12 weeks of intervention at a specialised laboratory. Serum glycated haemoglobin concentration (%) was determined by turbidimetry, and total cholesterol (mg/dl), triglycerides (mg/dl), high-density lipoprotein cholesterol (HDL-c, mg/dl) and low-density lipoprotein cholesterol (LDL-c, mg/dl) were determined by an enzymatic colorimetric method. Glutathione peroxidase (μmol/l) was quantified by an enzymatic method and CRP (mg/dl) was evaluated by turbidimetry. All exams were performed using commercials kits.

### Diagnosis of PE

PE was diagnosed by the medical team based on: the gradual development of hypertension (after the 20th gestational week) and the presence of proteinuria (>0⋅3 g protein excretion in 24 h urine), or chronic hypertension associated with proteinuria^(36)^. Blood pressure was measured by the nursing team and blood pressure variation was calculated as the difference between blood pressure measured at the first and last prenatal nutritional appointment.

The risk of developing early PE, up to the 34th gestational week, was estimated using the algorithm created and published by the Fetal Medicine Foundation^(37)^, version 2.5.0. The cut-off adopted to classify pregnancies at high risk of early PE was when it was greater than 1:200^(38)^.

### Sociodemographic, biological and obstetric assessment

The characteristics evaluated were: partnership status (partnered/unpartnered), maternal age (years) skin colour (by self-classification – white/black or brown), household income (in multiples of the minimum wage, based on its 2019 value) and educational level (high school non-graduate/high school graduate or higher education). As for the patients’ obstetric history, parity (0/≥1) and personal history of GHS (yes/no) were obtained.

The biological characteristics collected were: type of DM, duration of DM and presence of chronic diseases (hypertension and/or thyroid disorders). In addition, prenatal care variables were observed: gestational age at the first prenatal appointment and the first prenatal nutritional appointment (weeks), the total number of prenatal appointments and prenatal nutritional appointments calcium supplements use (yes/no) and basal insulin use.

### Outcomes

The primary outcome was the PE incidence (%). The secondary outcomes included systolic and diastolic blood pressure levels (mmHg), changes in glycated haemoglobin (%), total cholesterol (mg/dl), LDL-c (mg/dl), HDL-c (mg/dl), triglycerides (mg/dl), CRP (mg/dl) and glutathione peroxidase (μmol/l). All secondary outcomes were compared between study groups (inter-group analysis) and were also compared within each group (intra-group analysis) and considered the means or medians variations between baseline and post-intervention.

### Sample size

The sample size was calculated based on the primary outcome (prevalence of hypertensive disorders of pregnancy) using G*Power^(39)^. The sample was calculated considering a type I error of 5 % (*α* = 0⋅05), 80 % power, an estimated prevalence of 25 % of PE in women with pre-existing DM^(40,41)^ and an effect size (*w*) of 0⋅5. Based on this calculation, a minimum sample of sixteen women was estimated for each group^(42)^. Considering the longitudinal design of the study and possible follow-up losses (around 20 %), the minimum sample size for each study group was set at twenty participants.

### Statistical analysis

Data distribution was evaluated by the Shapiro–Wilk test and visual inspection. The sample was described using mean and standard deviation, median and interquartile range, or relative and absolute frequencies. To identify differences in the general characteristics and dietary intake of the groups, the Student's *t* test or the Mann–Whitney *U* test was used.

The PE incidence (primary outcome) and categorical variables were compared between the groups using the χ^2^ test or Fisher's exact test, if necessary.

Student's *t* test or the Mann–Whitney *U* test were also used to compare the means or medians variations in metabolic outcomes (secondary outcomes) in inter-group analysis, while the paired-sample Student's *t* test or Wilcoxon test was used to assess variations intra-group. Significance was set at 5 % and a 95 % confidence interval (CI) was calculated. Nutrient intake was described for energy-adjusted data by the energy density method^(43)^. Analyses were performed using SPSS Statistics 21.0 (IBM).

### Ethical issues

All the women in the study signed an informed consent form and the project was approved by the ME/UFRJ research ethics committee on 07/31/15 (CAAE 47335515.0.0000.5275). The study was registered in the Brazilian Clinical Trials Registration Platform (Rebec – RBR-4tbgv6) and was conducted according to the guidelines laid down in the Declaration of Helsinki.

## Results

Eighty-seven women were initially enrolled for the study between 2016 and 2020: forty were allocated to the DDG, forty-seven to the SDG, and there were nineteen follow-up losses (twelve from the SDG and seven from the DDG): five due to a change in prenatal care provider; three who discontinued the intervention; two who were diagnosed with latent autoimmune diabetes in adults and maturity-onset diabetes of the young; eight because of COVID-19 lockdown measures and one who received a diagnosis of syphilis (Fig. 1).

Gestational age at the beginning of treatment varied between participants depending on the start of prenatal care at the facility, but no statistically significant difference was observed between the intervention groups (SDG: 14 [IQR = 11, 20] *v*. DDG: 16 [IQR = 11, 18] weeks, *P* = 0⋅39 – data not showed in tables). The duration of the intervention also varied between participants (total mean = 17⋅9 weeks; sd = 6⋅7), with no statistically significant differences between groups (Table 1).

**Table 1.** Characteristics of the study participants according to study group (Brazil, Rio de Janeiro, 2016–2020)

Variables	SDG *n* (%)	DDG *n* (%)	*P*
Marital status (*n* 68)			
Lives without a partner	6 (17⋅1)	7 (21⋅2)	0⋅67^a^
Lives with a partner	29 (82⋅9)	26 (78⋅8)	
Family income (*n* 64)			
<Minimum wage	4 (11⋅4)	2 (6⋅9)	0⋅68^b^
≥Minimum wage	31 (88⋅6)	27 (93⋅1)	
Skin colour (*n* 67)			
White	9 (26⋅5)	11 (33⋅3)	0⋅54^a^
Brown/Black	25 (73⋅5)	22 (62⋅1)	
Education level (*n* 68)			
No complete high school	13 (37⋅1)	10 (30⋅3)	0⋅29^a^
Complete high school	22 (62⋅9)	23 (69⋅7)	
Diabetes, type (*n* 68)			
Type 1	16 (45⋅7)	17 (51⋅5)	0⋅63^a^
Type 2	19 (54⋅3)	16 (48⋅5)	
Preeclampsia, risk (*n* 35)			
Low, risk	12 (60⋅0)	8 (40⋅0)	0⋅24^a^
High, risk	6 (40⋅0)	9 (60⋅0)	
Pre-gestational weight status (*n* 68)			
Eutrophic	12 (34⋅3)	7 (21⋅2)	0⋅23^a^
Overweight or Obesity	23 (65⋅7)	26 (78⋅8)	
Parity (*n* 68)			
0	12 (34⋅3)	12 (36⋅4)	0⋅86^a^
≥1	23 (65⋅7)	21 (63⋅6)	
GHS, history (*n* 42)			
Yes	3 (13⋅6)	4 (20⋅0)	0⋅44^b^
No	19 (86⋅4)	16 (80⋅0)	
Presence of hypothyroidism (*n* 68)			
Yes	1 (2⋅9)	6 (18⋅2)	0⋅05^b^
No	34 (97⋅1)	27 (81⋅8)	
Presence of chronic arterial hypertension (*n* 68)			
Yes	5 (14⋅3)	7 (21⋅2)	0⋅45^a^
No	30 (85⋅7)	26 (78⋅8)	
Aspirin, use (*n* 68)			
Yes	28 (80⋅0)	30 (90⋅9)	0⋅31^b^
No	7 (20)	3 (9⋅1)	
Calcium supplement, use (*n* 68)			
Yes	4 (11⋅4)	7 (21⋅2)	0⋅27^a^
No	31 (88⋅6)	26 (78⋅8)	
Dietary method (*n* 68)			
Traditional	28 (80⋅0)	28 (84⋅8)	0⋅60^a^
Carbohydrate counting	7 (20⋅0)	5 (15⋅2)	
Variation in basal insulin dose during the study* (*n* 67)	33⋅5 (29⋅6)	29⋅2 (30⋅2)	0⋅56^c^
Intervention time, weeks* (*n* 68)	18⋅1 (6⋅7)	17⋅7 (7⋅1)	0⋅82^c^

SDG, standard diet group; DDG, DASH diet group; *n*, sample, GHS, gestational hypertensive syndrome.

a Chi-square test.

b Fisher's exact test.

c Student's *t* test.

* Values expressed as mean and standard deviation.

The median age of the study participants was 32 years (IQR = 26, 36), and there was no statistically significant difference in median age between the groups (SDG: 32 years [IQR = 26, 35] *v.* DDG: 32 years [IQR = 27, 37], *P* = 0⋅86). No statistically significant difference was found between the groups for the mean number of prenatal appointments (SDG: 12 [sd = 3] *v.* DDG: 12 [sd = 4], *P* = 0⋅89) and the median number of nutritional appointments (SDG: 6 [IQR = 5, 6] *v.* DDG: 6 [IQR = 5, 6], *P* = 0⋅76). There were no statistically significant differences between the groups for the mean duration of DM (DDG = 9 years [sd = 7]; SDG mean = 8 years [sd = 8]) – data not shown in tables. The socioeconomic and demographic characteristics and biological conditions of the two groups were similar, as were the proportions of women with T1DM and T2DM (Table 1).

No difference was observed in the incidence of PE between the groups, although there were a greater absolute proportion of cases in the SDG. The total incidence of PE in the study population was 17⋅7 % (Table 2).

**Table 2.** Preeclampsia incidence and adherence to diet according to study group (Brazil, Rio de Janeiro, 2016–2020)

Variables	SDG *n* (%)	DDG *n* (%)	*P*
Preeclampsia			
Yes	8 (22⋅9)	4 (12⋅1)	0⋅25^a^
No	27 (77⋅1)	29 (87⋅9)	
Adherence to the diet at the 2nd appointment (*n* 68)			
Poor	19 (54⋅3)	16 (48⋅5)	0⋅63^a^
Good/great	16 (45⋅7)	17 (51⋅5)	
Adherence to the diet at the 3rd appointment (*n* 68)			
Poor	20 (57⋅1)	19 (57⋅6)	0⋅97^a^
Good/great	15 (42⋅9.)	14 (42⋅4)	
Adherence to the diet at the 4th appointment (*n* 64)			
Poor	19 (57⋅6)	18 (58⋅1)	0⋅97^a^
Good/great	14 (42⋅4)	13 (41⋅9)	
Adherence to the diet at the 5th appointment (*n* 55)			
Poor	14 (46⋅7)	11 (44⋅0)	0⋅84^a^
Good/great	16 (53⋅3)	14 (56⋅0)	
Adherence to the diet at the 6th appointment (*n* 36)			
Poor	10 (50⋅0)	5 (31⋅3)	0⋅26^a^
Good/great	10 (50⋅0)	11 (68⋅8)	

SDG, standard diet group; DDG, DASH diet group; *n*, sample.

a Chi-square test.

Regarding diet adherence, there was no significant difference between the groups at any of the nutritional appointments (Table 2). The proportion of women with good or excellent adherence between the 2nd and 6th appointments ranged from 45⋅7 to 50⋅0 % in the SDG and between 51⋅5 and 68⋅8 % in the DDG. Regarding food intake, the women in the DDG had a statistically higher median consumption of omega-3 fatty acids than those in the SDG (Table 3).

**Table 3.** Dietary intakes of study participants during the study by nutrient density and (*n* 54)

Variables	Standard diet group (*n* 30)		DASH diet group (*n* 24)		
	Median	IQR	Median	IQR	*P*^a^
Energy (kcal/d)	2035⋅0	1721⋅2–2190⋅3	1934⋅5	1621⋅7–2036⋅2	0⋅27
Protein (g/1000 kcal)	47⋅9	41⋅4–52⋅0	48⋅8	46⋅55⋅4	0⋅33
Total, Fat (g/1000 kcal)	34⋅1	29⋅6–36⋅1	34⋅0	31⋅7–35⋅5	0⋅65
Cholesterol (mg/1000 kcal)	133⋅7	102⋅5–222⋅6	161⋅7	113⋅9–229⋅3	0⋅16
Omega 3 – fatty acids (g/1000 kcal)	0⋅9	0⋅7–1⋅0	1⋅0	1⋅0–1⋅6	0⋅04
Sodium (mg/1000 kcal)	675⋅9	550⋅1–747⋅1	603⋅1	499⋅7–844⋅3	0⋅64
Selenium (mg/1000 kcal)	51⋅2	42⋅1–67⋅0	60⋅5	51⋅9–78⋅3	0⋅06
	Mean	sd	Mean	sd	*P*^b^
Carbohydrate (g/1000 kcal)	127⋅6	14⋅2	121⋅6	13⋅0	0⋅11
Fibre (g/1000 kcal)	16⋅0	4⋅1	16⋅3	4⋅0	0⋅82
Saturated fatty acid (g/1000 kcal)	10⋅6	2⋅4	10⋅1	2⋅2	0⋅47
Polyunsaturated fatty acid (g/1000 kcal)	8⋅9	2⋅9	9⋅9	2⋅1	0⋅16
Monounsaturated fatty acid (g/1000 kcal)	9⋅8	2⋅29	10⋅1	2⋅2	0⋅57
Calcium (mg/1000 kcal)	524⋅9	168⋅0	608⋅3	219⋅2	0⋅12
Potassium (mg/1000 kcal)	1487⋅9	310⋅7	1596⋅5	363⋅1	0⋅24
Magnesium (mg/1000 kcal)	148⋅7	27⋅9	161⋅0	34⋅6	0⋅16

*n*, sample; IQR, Interquartile range; sd, Standard deviation.

a Obtained from U – Mann–Whitney *U* test.

b Obtained from Student's *t* test.

The first assessment of metabolic markers (baseline) was done at a mean gestational age of 17 (sd = 6) weeks and the post-intervention evaluation was done at a mean gestational age of 29 (sd = 5) weeks. In the intra-group variation during the study, for the SDG there was an average increase in total cholesterol (32 [CI  16, 48], *P* = 0⋅00), LDL-cholesterol (17 [CI  6, 28], *P* = 0⋅00) and triglycerides (42⋅8 [CI  27⋅1, 58⋅6], *P* = 0⋅00). There was a reduction in glycated haemoglobin (−0⋅6 [CI  −0⋅96, −0⋅26], *P* = 0⋅00) and there were no changes in systolic and diastolic blood pressure, HDL-cholesterol, CRP and glutathione peroxidase (Table 4).

**Table 4.** Characteristics of metabolic outcomes at baseline and after intervention (Rio de Janeiro, 2016–2020)

Outcomes	Standard diet group								DASH diet group								
	Baseline		Post-intervention		Change		*n*	*P*^†^	Baseline		Post-intervention		Change		*n*	*P*^†^	*P*^‡^
	Mean	sd	Mean	sd	Mean	sd			Mean	sd	Mean	sd	Mean	sd			
Glycated Haemoglobin (%)	7⋅1	1⋅1	6⋅5	0⋅8	−0⋅6	0⋅9	30	0⋅00^a^	7⋅3	1⋅7	6⋅2	0⋅9	−1⋅1	1⋅4	29	0⋅00^a^	0⋅12^b^
Glutathione peroxidase (μmol/l)	8092	1934	8121	2442	30	1987	11	0⋅96^a^	6633	2476	8221	3152	1588	2328	13	0⋅03^a^	0⋅09
Total, cholesterol (mg/dl)	182	28	214	42	32	33	20	0⋅00^a^	185	39	210	48	25	24	18	0⋅00^a^	0⋅48^c^
LDL-cholesterol (mg/dl)	89⋅0	25	106	38	17	26	22	0⋅00^a^	97	34	107	36	10	33	18	0⋅21^a^	0⋅43^c^
HDL-cholesterol (mg/dl)	67	19	67	19	−0⋅5	13	22	0⋅87^a^	72	19	70	18	2	10	18	0⋅44^a^	0⋅71^c^
Triglycerides (mg/dl)	125	54	168	55	43	35	21	0⋅00^a^	111	42	164	51	53	39	18	0⋅00^a^	0⋅40^c^
CRP (mg/dl)	0⋅8	0⋅5	1⋅1	1⋅1	0⋅3	1⋅0	16	0⋅29^a^	1⋅3	0⋅7	1⋅2	0⋅9	−0⋅1	0⋅5	12	0⋅49^a^	0⋅09^b^
	**Standard diet group**				*P*^†^				**DASH diet group**				*P*^†^				*P*^‡^
	Median, IQR								Median, IQR								
Systolic blood pressure (mmHg), baseline	117				–				110				–				–
	100–121								100–120								
Systolic blood pressure (mmHg), post-intervention	120				0.14^d^				120				0.46^d^				0.65^b^
	110–120								110–120								
Diastolic blood pressure (mmHg) in baseline	70				–				70				–				–
	60–80								70–80								
Diastolic blood pressure (mmHg)^,^ post-intervention	73				0.08^d^				70				0.65^d^				0⋅36^b^
	70–81								70–80								

CRP, C-reactive protein.

a Student's *t* test for paired samples.

b Mann–Whitney *U* test.

c Student's *t* test for independent samples.

d Wilcoxon test for comparison intra-group.

^*^Blood pressure values measured at the first and last prenatal nutritional care consultation expressed in median and interquartile range.

† Comparison intra-group.

‡ Comparison of changes between the DASH diet group and the standard diet group.

In the analysis of intra-group variation during the study, there was for the DDG an average increase in glutathione peroxidase (1588 [CI  181, 2994], *P* = 0⋅03), total cholesterol (25 [CI  13, 37], *P* = 0⋅00) and triglycerides (53 [CI  34, 72], *P* = 0⋅00). There was a reduction in glycated haemoglobin (−1⋅1 [CI  −1⋅6, −0⋅6], *P* = 0⋅00), and there were no changes in systolic and diastolic blood pressure, HDL-cholesterol and CRP (Table 4).

No significant differences occurred in the inter-group analysis. Glycated haemoglobin serum concentrations decreased in the same way in both groups and the DDG showed a significant increase in glutathione peroxidase which tended to be different between SDG and DDG (Table 4).

## Discussion

No differences between the group that received the standard diet and the group that received the adapted DASH diet were observed for the incidence of PE during the intervention. DDG showed an increase in serum concentrations of glutathione peroxidase and both diets contributed to a reduction of glycated haemoglobin. As the authors are unaware of any previous research evaluating the effect of the DASH diet on pregnant women with PDM, the discussion draws on the findings of studies with pregnant women with some other risk factors for PE.

The incidence of total PE in this study was 17⋅7 %. According to Persson *et al.*^(41)^, the incidence of PE is two to six times higher among women with PDM than among healthy pregnant women, and the risk is 2⋅5 times higher among women with pre-existing T1DM than among those with pre-existing T2DM. Compared to the study by Rezende *et al.*^(44)^ with healthy Brazilian women admitted for delivery at the same maternity hospital as this study, the incidence of PE in this study was 2⋅6 times higher.

In the present study, the adapted DASH diet was not associated with any improvement in blood pressure or incidence of PE, which corroborates the findings of Fulay *et al.*^(45)^, who investigated the impact of this diet on hypertensive disorders of pregnancy (chronic hypertension, gestational hypertension and preeclampsia) in 1760 American women. But contradicts those of Asemi *et al.*^(42)^, who demonstrated the effect of the DASH diet in reducing systolic blood pressure in Iranian women with GDM. Similarly, Jiang *et al.*^(20)^ found a lower incidence of PE among Chinese women with gestational hypertension or chronic hypertension who observed the DASH diet compared to those on a control diet. However, partial adherence to the diets may have contributed to our findings.

Studies have found that women with DM and PE have higher serum glycated haemoglobin concentrations during pregnancy^(46,47)^. Both groups presented average values within those recommended by the American Diabetes Association and the Brazilian Diabetes Society^(7,8)^, which both recommend levels of 6 % and up to 7 % to prevent hypoglycaemic episodes. However, only in the DDG was there a mean reduction of 1 % in glycated haemoglobin, which is considered a protective reduction for the development of PE^(8,46)^. The behaviour of glycated haemoglobin seen in the DDG may have been due to the overall glycaemic load^(5)^, since the glycaemic index of the DASH diet is low^(14)^, and the higher consumption of omega-3 fatty acids, which have an anti-inflammatory effect^(48)^.

The adapted DASH diet in this study had no effect on serum lipid concentrations, unlike in the women with GDM studied by Asemi *et al.*^(42)^. Nevertheless, LDL-c did not increase in the DDG, while it did in the SDG. This finding is relevant since LDL-c has pro-inflammatory action on the body and is associated with endothelial dysfunction, which contributes to the development and severity of PE^(49–51)^. In a study involving 240 pregnant women from Nigeria, Olalere *et al.*^(52)^ identified increased serum cholesterol and triglyceride and a positive correlation between PE and all lipid fractions. Notably, in both groups of this research, the total cholesterol and LDL-c variations throughout the study were smaller than those observed in other studies with pregnant women who did not receive prenatal nutritional care^(53,54)^.

Regarding the oxidative stress marker, the DDG group had increased glutathione peroxidase and a tendency to reduce levels of the inflammatory marker CRP, which suggests improved control of oxidative stress. According to Taravati and Tohdini^(55)^, oxidative stress products are involved in the endothelial dysfunction that occurs in PE for reasons not yet clarified.

The nutritional intake of the women on the DASH diet described by Asemi *et al.*^(42)^ included 17⋅6 % total fat and 3⋅5 % saturated fat, differing significantly from the dietary intake of the Brazilian women, who consumed an average of 29⋅6 % lipids and 9⋅4 % saturated fats^(56)^. The fat consumption of the women in the study by Asemi *et al.*^(42)^ was very different from the original DASH diet, which proposes 27 % lipids and 6 % saturated fat^(57)^, which may explain the results of their study. However, since the Brazilian population tends to have a high protein intake, including animal proteins, it is difficult to compare the findings with those of Asemi *et al.*^(42,56)^.

Interestingly, the food intake of the research participants from both groups met the Brazilian Diabetes Society recommendations^(7)^ for all nutrients, including saturated fat. The consumption of some nutrients was higher than has been described for Brazilian women of childbearing age and in studies evaluating the dietary intake of pregnant women without prenatal nutritional care. Compared to other studies with Brazilian pregnant women^(58,59)^, in the present study, the average intake of calcium, potassium and magnesium was higher for both groups, while sodium intake was lower, suggesting that prenatal nutritional care has a positive impact on diet. Previous studies of women who developed GDM have identified the protective role of regular prenatal nutritional care with a minimum of six appointments on foetal macrosomia and glycaemic profile^(21,60)^.

The modest findings of this research could be attributable to the difference between the metabolic conditions of the population under study and pregnant women with GDM or hypertensive disorders and the variations in dietary adherence observed in both groups throughout the study, resulting in a similar nutritional intake, not to mention the influence of Brazilian dietary habits. Further, both groups received prenatal care at a referral hospital for mid- to high-risk pregnancies from a multidisciplinary team which involved regular medical appointments and a minimum of six prenatal appointments with a nutritionist, meaning that both study groups were given guidance in line with the dietary recommendations of the Brazilian Society of Diabetes.

To date, no studies have verified the effect of the DASH diet on the prevention of PE in pregnant women with PDM, making this research unique in its scope. One limitation was the sample size, which was not large enough to ascertain the effects of the DASH diet and the fact that a private laboratory outside the study site was used to collect the blood for the analysis of the biochemical parameters, constituting a difficulty in obtaining more samples for analysis. Furthermore, the study period coincided with the COVID-19 pandemic, which meant data collection had to be interrupted due to the need for social isolation. The population of pregnant women with PDM is very heterogeneous concerning the type of diabetes and the presence of comorbidities, so it was not possible to conduct separate analyses for the women with T1DM and T2DM.

The adoption of the standard diet or adapted DASH diet, together with multidisciplinary prenatal care, seems to have resulted in adequate glycaemic control, with no difference in the incidence of PE, considering partial adherence to diets. Nonetheless, the adapted DASH diet seems to favour biochemical markers related to oxidative stress, which are associated with the development of PE. This finding suggests that the adapted DASH diet may be an option for pregnant women with PDM who have other risk factors for cardiovascular disease in addition to chronic hypertension. However, further studies are needed with larger sample sizes to confirm these results.
