# Effect of the Dietary Approaches to Stop Hypertension (DASH) diet on the development of preeclampsia and metabolic outcomes in pregnant women with pre-existing diabetes mellitus: a randomized, controlled, single-blind trial – CORRIGENDUM

**DOI:** 10.1017/jns.2023.77

**Published:** 2023-07-28

**Authors:** Gabriella P. Belfort, Patrícia de Carvalho Padilha, Dayana R. Farias, Letícia B. G. da Silva, Karina dos Santos, Erlaine de S. Gomes, Thaissa S. V. Lima, Rita Bernardete R. G. Bornia, Karina B. C. Rezende, Claudia Saunders

**Affiliations:** 1 Josué de Castro Institute of Nutrition, Federal University of Rio de Janeiro, 373, Carlos Chagas Filho Ave, University City, Rio de Janeiro, RJ 21941-590, Brazil; 2 Applied Nutrition Department, Federal University of the State of Rio de Janeiro, 296, Pasteur Ave, Rio de Janeiro, RJ 22290-240, Brazil; 3 Social and Applied Nutrition Department, Josué de Castro Institute of Nutrition, Federal University of Rio de Janeiro, 373, Carlos Chagas Filho Ave, University City, Rio de Janeiro, RJ 21941-590, Brazil; 4 Public Health Nutrition Department, Federal University of the State of Rio de Janeiro, 296, Pasteur Ave, Rio de Janeiro, RJ 22290-240, Brazil; 5 Maternity School of the Federal University of Rio de Janeiro, 180 Laranjeiras St, Rio de Janeiro, RJ, 22240-003, Brazil

Details: Correct author name on title page

Currently reads: Patricia C de Padilha

This should read: Patrícia de Carvalho Padilha

